# Retrospective survey of *Dickeya fangzhongdai* using a novel validated real-time PCR assay

**DOI:** 10.3389/fmicb.2023.1249955

**Published:** 2024-02-13

**Authors:** Špela Alič, Katarina Bačnik, Tanja Dreo

**Affiliations:** National Institute of Biology, Ljubljana, Slovenia

**Keywords:** molecular testing, diagnostics, plant pathogen, real-time PCR, *Dickeya*, survey, water

## Abstract

*Dickeya fangzhongdai*, an aggressive plant pathogen, causes symptoms on a variety of crops and ornamental plants including bleeding canker of Asian pear trees. Historical findings stress the need for a specific detection tool for *D. fangzhongdai* to prevent overlooking the pathogen or assigning it to general *Dickeya* spp. Therefore, a qualitative real-time PCR for specific detection of *D. fangzhongdai* has been developed and validated. The developed assay shows selectivity of 100%, diagnostic sensitivity of 76% and limit of detection with 95% confidence interval in plant matrices ranging from 311 to 2,275 cells/mL of plant extracts. The assay was successfully used in a retrospective survey of selected host plants of relevance to Europe and environmental niches relevant to *D. fangzhongdai*. Samples of potato tubers and plants, plants from the *Malinae* subtribe (apple, pear, quince, and Asian pear tree) and fresh surface water from Slovenia were analyzed. *D. fangzhongdai* was not detected in any plant samples, however, 12% of surface water samples were found to be positive.

## 1 Introduction

*Dickeya fangzhongdai* is one of the more recently described species of the pectinolytic genus *Dickeya* (Tian et al., 2016). This genus groups diverse bacterial isolates that cause soft-rot disease in a variety of plant species, including economically important crops and ornamental plants, and cause wilting, black leg, and soft-rot symptoms (Toth et al., 2011). Reports of soft-rot disease caused by the genus *Dickeya* have been limited to herbaceous plants, however, *D. fangzhongdai* was originally described as a causative agent of bleeding canker of Asian pear *(Pyrus pyrifolia)* in China (Tian et al., 2016). The pathogen description was later expanded to soft rot strains isolated from various plant species, prevalently in monocots (Alič et al., 2017a,^2018^), and isolates from surface water (Pritchard et al., 2013a; Alič et al., 2018). Since the description of the species, multiple reports of the pathogen have been made in various host plants (see Table 1), including affecting fruits of additional tree species (Jaffar et al., 2019). The species description has also been expanded to include strains isolated in the last century. Extension of the host range of *Dickeya* spp. to trees is a relatively new observation, however, it does not appear to be a unique characteristic of *D. fangzhongdai*, since it was also reported for *D. dadantii* (Ogoshi et al., 2019; Fujikawa et al., 2020). Both species predominantly affect fruit trees such as pear, apple, peach, and jackfruit trees (Tian et al., 2016; Ogoshi et al., 2019; Chen et al., 2020; Fujikawa et al., 2020; Choi et al., 2021). Based on the literature, the majority of the reported *D. fangzhongdai* strains and all tree infections have been reported in Asian countries (Table 1). There is little information regarding the economic damage and the extent of bacterial occurrence in host plants outside of Asia. The bacteria were reported as a causative agent of soft rot of orchids in commercial production in Europe (Alič et al., 2017a) and Canada (Zhou et al., 2021), which originated with material from Asia and Europe, and as the causative agent of soft rot of onions in USA (Ma et al., 2020). However, based on the outbreak reports, it has a more significant impact on agriculture in Asia. While data on losses is limited, several authors report outbreaks on various economically important plants such as orchids (Shen et al., 2019; Balamurugan et al., 2020; Wei et al., 2021; Chi et al., 2022), banana (Yang et al., 2022), onion (Tsai et al., 2019; Wei et al., 2021), jack fruit (Jaffar et al., 2019), Asian pear (Tian et al., 2016; Choi et al., 2021), and even staple food crops such as taro (Dobhal et al., 2020; Huang et al., 2021; Hugouvieux-Cotte-Pattat et al., 2022). Based on the reports, it can be surmised that *D. fangzhongdai* is well established in Asia.

**TABLE 1:** Reports of *D. fangzhongdai* species in the literature to date. Most of the reported *D. fangzhongdai* strains were isolated in Asia.

*D. fangzhongdai* strain	GenBank accession^1^	Host^2^	Origin^2^	Year of isolation	References^3^
DSM 101947 (JS5)^T^	CP025003 ^4^	*Pyrus pyrifolia*	China	2009–2010	Tian et al., 2020
LN1	CP031505 ^4^	*Pyrus pyrifolia*	China	2009–2010	Tian et al., 2020
QZH3	CP031507 ^4^	*Pyrus pyrifolia*	China	2009–2010	Tian et al., 2020
ECM-1	MT820458	*Pyrus pyrifolia*	Korea	2019	Choi et al., 2021
ECM-2	MT820459	*Pyrus pyrifolia*	Korea	2019	Choi et al., 2021
ECM-3	MT820460	*Pyrus pyrifolia*	Korea	2019	Choi et al., 2021
B16	CP087226 ^4^	*Phalaenopsis* sp.	Slovenia	2010	Alič et al., 2017a
S1	JXBO00000000 ^4^	*Phalaenopsis* sp.	Slovenia	2012	Alič et al., 2017a
FSPAD1	MK394174	*Phalaenopsis aphrodite*	China	2017	Shen et al., 2019
Kot1	MN400213	*Dendrobium nobile*	India	2018	Balamurugan et al., 2020
Kot2	MN400214	*Dendrobium nobile*	India	2018	Balamurugan et al., 2020
Kot5	MN400217	*Dendrobium nobile*	India	2018	Balamurugan et al., 2020
Ph1	MZ081223	*Phalaenopsis* sp.	Taiwan	NA	Wei et al., 2021
Ph2	MZ081209	*Phalaenopsis* sp.	Taiwan	NA	Wei et al., 2021
Ph3	MZ081210	*Phalaenopsis* sp.	Taiwan	NA	Wei et al., 2021
Ph20	MZ081228	*Phalaenopsis* sp.	Taiwan	NA	Wei et al., 2021
Ph21	MZ081208	*Phalaenopsis* sp.	Taiwan	NA	Wei et al., 2021
Ph22	MZ081211	*Phalaenopsis* sp.	Taiwan	NA	Wei et al., 2021
Ph29	MZ081212	*Phalaenopsis* sp.	Taiwan	NA	Wei et al., 2021
Ph16	MZ081224	*Phalaenopsis* sp.	Taiwan	NA	Wei et al., 2021
Ph17	MZ081225	*Phalaenopsis* sp.	Taiwan	NA	Wei et al., 2021
Ph18	MZ081226	*Phalaenopsis* sp.	Taiwan	NA	Wei et al., 2021
Ph19	MZ081227	*Phalaenopsis* sp.	Taiwan	NA	Wei et al., 2021
Ph4	MZ081204	*Phalaenopsis* sp.	Taiwan	NA	Wei et al., 2021
Ph5	MZ081202	*Phalaenopsis* sp.	Taiwan	NA	Wei et al., 2021
Ph6	MZ081213	*Phalaenopsis* sp.	Taiwan	NA	Wei et al., 2021
Ph7	MZ081214	*Phalaenopsis* sp.	Taiwan	NA	Wei et al., 2021
Ph8	MZ081215	*Phalaenopsis* sp.	Taiwan	NA	Wei et al., 2021
Ph9	MZ081216	*Phalaenopsis* sp.	Taiwan	NA	Wei et al., 2021
Ph10	MZ081205	*Phalaenopsis* sp.	Taiwan	NA	Wei et al., 2021
Ph11	MZ081217	*Phalaenopsis* sp.	Taiwan	NA	Wei et al., 2021
Ph12	MZ081200	*Phalaenopsis* sp.	Taiwan	NA	Wei et al., 2021
Ph13	MZ081218	*Phalaenopsis* sp.	Taiwan	NA	Wei et al., 2021
Ph14	MZ081203	*Phalaenopsis* sp.	Taiwan	NA	Wei et al., 2021
Ph15	MZ081201	*Phalaenopsis* sp.	Taiwan	NA	Wei et al., 2021
Ph23	MZ081229	*Phalaenopsis* sp.	Taiwan	NA	Wei et al., 2021
Ph24	MZ081221	*Phalaenopsis* sp.	Taiwan	NA	Wei et al., 2021
Ph25	MZ081198	*Phalaenopsis* sp.	Taiwan	NA	Wei et al., 2021
Ph26	MZ081199	*Phalaenopsis* sp.	Taiwan	NA	Wei et al., 2021
Ph27	MZ081220	*Phalaenopsis* sp.	Taiwan	NA	Wei et al., 2021
Ph28	MZ081222	*Phalaenopsis* sp.	Taiwan	NA	Wei et al., 2021
VNO1 (LC.H1)	MW281723	*Paphiopedilum concolor*	Vietnam	2020	Chi et al., 2022
VNO2 (YB.H6)	MW281724	*Paphiopedilum concolor*	Vietnam	2020	Chi et al., 2022
VNO3 (LS.HD9)	MW281726	*Phalaenopsis amabilis*	Vietnam	2020	Chi et al., 2022
VNO4 (TN.PD11)	MW281727	*Dendrobium anosmum*	Vietnam	2020	Chi et al., 2022
VNO5 (HN.PD16)	MW281728	*Dendrobium anosmum*	Vietnam	2020	Chi et al., 2022
VNO6 (SL.PD20)	MW281729	*Dendrobium anosmum*	Vietnam	2020	Chi et al., 2022
VNO7 (QN.PD24)	MW281730	*Dendrobium anosmum*	Vietnam	2020	Chi et al., 2022
VNO8 (GL.PD26)	MW281731	*Dendrobium anosmum*	Vietnam	2020	Chi et al., 2022
VNO9 (BP.HD29)	MW281732	*Phalaenopsis amabilis*	Vietnam	2020	Chi et al., 2022
908C	JADCNJ00000000 ^4^	Orchid	Canada	2020	Zhou et al., 2021
ZXC1	MN853405	*Colocasia esculenta*	China	NA	Huang et al., 2021
MPC2	MN853406	*Colocasia esculenta*	China	NA	Huang et al., 2021
Orc3-1	MT613404	*Colocasia esculenta*	Taiwan	NA	Huang et al., 2020 (GenBank submission)
Orc6-2	MT613403	*Colocasia esculenta*	Taiwan	NA	Huang et al., 2020 (GenBank submission)
M1L2b-2	MT613402	*Colocasia esculenta*	Taiwan	NA	Huang et al., 2020 (GenBank submission)
TH11	MT613401	*Colocasia esculenta*	Taiwan	NA	Huang et al., 2020 (GenBank submission)
M1L1	MT613400	*Colocasia esculenta*	Taiwan	NA	Huang et al., 2020 (GenBank submission)
C2	MT613399	*Colocasia esculenta*	Taiwan	NA	Huang et al., 2020 (GenBank submission)
M1D3-2	MT613398	*Colocasia esculenta*	Taiwan	NA	Huang et al., 2020 (GenBank submission)
20-1	MT613397	*Colocasia esculenta*	Taiwan	NA	Huang et al., 2020 (GenBank submission)
M1O1-2	MT613396	*Colocasia esculenta*	Taiwan	NA	Huang et al., 2020 (GenBank submission)
M1A1-2	MT613395	*Colocasia esculenta*	Taiwan	NA	Huang et al., 2020 (GenBank submission)
918-9-1	MT613394	*Colocasia esculenta*	Taiwan	NA	Huang et al., 2020 (GenBank submission)
918-8-2	MT613393	*Colocasia esculenta*	Taiwan	NA	Huang et al., 2020 (GenBank submission)
918-9-2	MT613392	*Colocasia esculenta*	Taiwan	NA	Huang et al., 2020 (GenBank submission)
B7-15 16S	MT613391	*Colocasia esculenta*	Taiwan	NA	Huang et al., 2020 (GenBank submission)
PL145	MN812278	*Colocasia esculenta*	Hawaii, USA	NA	Dobhal et al., 2020
PL146	MN812277	*Colocasia esculenta*	Hawaii, USA	NA	Dobhal et al., 2020
NCPPB 2929	MZ611617	*Colocasia esculenta*	Solomon Islands	NA	Hugouvieux-Cotte-Pattat et al., 2022
NCPPB 3274	CM001979 ^4^	*Aglaonema*	St. Lucia	1983	Pritchard et al., 2013a
643b	CP092458 ^4^	*Aglaonema* sp.	USA	2020	Asselin et al., 2022 (GenBank submission)
CAS9	MZ081207	*Allium fistulosum*	Taiwan	NA	Wei et al., 2021
IAS4	MZ081219	*Allium fistulosum*	Taiwan	NA	Wei et al., 2021
TAS1	MZ081206	*Allium fistulosum*	Taiwan	NA	Wei et al., 2021
N1	MK256333	*Allium fistulosum*	Taiwan	2018	Tsai et al., 2019
AP6	CP092460 ^4^	*Allium cepa*	USA	2014	Ma et al., 2020
GZF2-2	MZ76892	*Musa* spp.	China	NA	Yang et al., 2022
GZF1-8	OK668082	*Musa* spp.	China	NA	Yang et al., 2022
ZG5	MW332472	*Pinellia ternata*	China	2020	Wang et al., 2021
MK7	CM001984 ^4^	River water	Scotland (UK)	NA	Pritchard et al., 2013a
ND14b^5^	CP009460 ^4^	Waterfall	Malaysia	2013	Chan, 2014 (GenBank submission)
M005^5^	JSXD00000000 ^4^	Waterfall	Malaysia	2013	Chan and Tan, 2014 (GenBank submission)
M074^5^	JRWY00000000 ^4^	Waterfall	Malaysia	2013	Chan and Tan, 2014 (GenBank submission)
GR29	MH429934	Estuarine water	India	2017	Khandeparker and Eswaran, 2018 (GenBank submission)
631d	MH842153	*Artocarpus heterophyllus*	Malaysia	2018	Jaffar et al., 2019
131	MH842152	*Artocarpus heterophyllus*	Malaysia	2018	Jaffar et al., 2019
241	MH197139	*Artocarpus heterophyllus*	Malaysia	2018	Jaffar et al., 2019
YZY-SG-17	MW160421	*Belamcandae* Rhizoma	China	NA	Liu, 2020 (GenBank submission)
Secpp 1600	CP023484 ^4^	Radish	China	2016	Cheng et al., 2017 (GenBank submission)
VNO2R	MW281725	NA	Vietnam	2020	Chi et al., 2022
Onc5	CP080400 ^4^	NA	China	2021	Pan, 2021 (GenBank submission)
IPO4215	OM809171	*Solanum tuberosum*	Netherlands	2020	van der Wolf et al., 2022
IPO4216	OM809172	*Solanum tuberosum*	Netherlands	2020	van der Wolf et al., 2022

^1^GenBank accession numbers are provided for genomic (whole genome data) sequences or, if those are not available, accession numbers for 16S ribosomal RNA gene, *dnaX*, *gap*A or other accessible gene are provided.

^2^Sample description for GenBank submissions were derived from the sample metadata or the submission title if metadata were not available.

^3^For the GenBank submissions the first author and the year of the submission is stated. Each GenBank submission is clearly noted by the term “GenBank submission” in the brackets.

^4^Genome sequence included in the ANI analysis.

^5^Strains were originally described as other species, but later identified as *D. fangzhongdai* (Alič et al., 2018).

There are currently no data on whether the strains causing bleeding canker can infect and cause soft rot symptoms of herbaceous plants in nature, however, based on inoculation experiments, pear isolates can cause symptoms on potato, tomato, cabbage, and orchids (Chen et al., 2020). The ability to persist in potato plants was also reported for the orchid pathogens *D. fangzhongdai* S1 and B16 (Alič et al., 2017a). In addition, *D. fangzhongdai* was isolated from field grown potato tubers in a 2020 study in the Netherlands. The isolates showed high virulence in a field bioassay, capable of causing blackleg to a similar extent as *Pectobacterium brasiliense* (van der Wolf et al., 2022). As yet, the species is not associated with diseases of food crops outside of Asia. However, the high aggressiveness of some *D. fangzhongdai* strains (Alič et al., 2017a; Chen et al., 2020; van der Wolf et al., 2022), the general adaptability of the genus *Dickeya* (Toth et al., 2011) and the plasticity of the species phenotype (Alič et al., 2018) call for caution.

Multiple *D. fangzhongdai* outbreaks (Tian et al., 2016; Jaffar et al., 2019; Shen et al., 2019; Tsai et al., 2019; Balamurugan et al., 2020; Ma et al., 2020; Choi et al., 2021; Zhou et al., 2021; Chi et al., 2022; Yang et al., 2022) in recent years suggest that the pathogen is present and spreading in Asia and also other continents. It was indicated before that trade plays can promote the spread of the *Dickeya* disease (Toth et al., 2011). Moreover, there is evidence that ornamental plants may represent potential routes for the introduction of additional *Dickeya* species and strains with a broad host range (Parkinson et al., 2009; Toth et al., 2011), as observed in *D. solani* spread to potato. Therefore, close consideration should be given to potential infection routes and the adaptability of these pathogens to other plant hosts and environments. Especially so for pathogens like *D. fangzhongdai* with a broad host range that predominantly consists of economically important plants with high trade rates (Hinsley et al., 2018). However, only adequately validated diagnostic tools enable timely identification of *D. fangzhongdai* infected plants to support prevention of its introduction and aid epidemiological investigations. Therefore, there is a great need for the development of specific and reliable diagnostic tools that enable monitoring the presence and spread of *D. fangzhongdai*. Two detection tests specific to *D. fangzhongdai* species were previously developed: a real-time PCR test described by Tian et al. (2020) specifically developed and tested on *D. fangzhongdai* strains from Asian pear trees, and a loop-mediated isothermal amplification (LAMP) test described by DeLude et al. (2022) that was comprehensively validated on taro, onion, and orchid matrices.

The aims of this study were to (i) develop a qualitative real-time PCR for specific detection of *D. fangzhongdai* in various plant matrices (and extend validation to novel matrices including potato and orchids) and ecological niches, (ii) to validate the developed test according to the guidelines of the European and Mediterranean Plant Protection Organization (EPPO) (European and Mediterranean Plant Protection organization, 2021), and compare its performance with the previously developed test described by Tian et al. (2020), and (iii) to use the newly developed test in a retrospective survey to assess the presence of these bacteria in potato plants with soft rot symptoms, and surface water in Slovenia.

## 2 Materials and methods

### 2.1 Selection of host plants and niches relevant for *D. fangzhongdai* survey

Based on previous experience with the occurrence of soft-rot and a literature search, an informed selection of potential target plants and environmental niches was made. The literature search was performed using Google Scholar, to find reports of novel *D. fanzhongdai* outbreaks and isolates. Further, the GenBank database (Sayers et al., 2022) was searched for *D. fanzhongdai* nucleotide sequences that have not yet been reported in any publication. Based on the search results, a table (Table 1) was created, summarizing the currently reported *D. fangzhongdai* strains, hosts, geographical origin, and year of isolation.

Average nucleotide identity (ANI) was calculated for all reported *D. fangzhongdai* isolates with publicly available genome sequences to confirm isolate identification using the Genome-based distance matrix calculator (Richter et al., 2016; Sayers et al., 2022).

### 2.2 Bacterial strains

Bacterial strains used in the study are shown in Table 2. *Dickeya* spp. were grown overnight on Casamino acid-Peptone-Glucose (CPG; Schaad et al., 2001) medium at 28°C. Bacteria of other genera were grown overnight on yeast-extract peptone glucose agar (YPGA; EU, 1993), CPG or nutrient agar (NA; Schaad et al., 2001) medium at 25°C.

**TABLE 2:** Table of bacteria isolates used to determine analytical specificity of the developed real-time PCR test.

Isolate	Host plant	Origin and year of isolation
**Target species**		
*Dickeya fangzhongdai*		
DSMS 101947 (JS5)^T^	*Pyrus pyrifolia*	China, 2009
B16	*Phalaenopsis* sp.	Slovenia, 2010
MK7	River water	Scotland, NA
NCPPB 3274	*Aglaonema* sp.	St. Lucia, 1983
S1	*Phalaenopsis* sp.	Slovenia, 2012
**Non-target species**		
*Dickeya solani*		
IPO 2222^T^	*Solanum tuberosum*	The Netherlands, 2007
RNS 08.23.3.1A	*Solanum tuberosum*	France, 2008
7044	NA	NA
GBBC 2040	*Solanum tuberosum*	Belgium, 2007
GBBC 500	*Solanum tuberosum*	Belgium, NA
GBBC 1021	*Solanum tuberosum*	Belgium, NA
*Dickeya dadantii* NCPPB 898	*Pelargonium capitatum*	Comoro Islands, 1961
*Dickeya dadantii* subsp. *dieffenbachiae* LMG 25992	*Dieffenbachia* sp.	USA, 1957
*Dickeya dianthicola*		
LMG 2485^T^	*Dianthus caryophyllus*	UK, 1956
8823	NA	NA
RNS 04.9	*Solanum tuberosum*	France, 2004
*Dickeya chrysanthemi*		
LMG 2804^T^	*Chrysanthemum morifolium*	USA, 1956
NCPPB 402	*Chrysanthemum morifolium*	USA, 1956
*Dickeya zeae*		
LMG 2497	*Zea mays* var. rugosa	USA, NA
LMG 2505^T^	*Zea mays*	USA, 1970
*Dickeya paradisiaca* LMG 2542	*Musa paradisiaca*	Colombia, 1973
*Dickeya aquatica* NCPPB 4589	River water	UK, 2008
*Pectobacterium wasabiae* LMG 25890	*Solanum tuberosum*	New Zealand, 2005
*Pectobacterium carotovorum* pv. *brasiliense* PRI 3710	NA	NA
*Pectobacterium atrosepticum* LMG 2386^T^	*Solanum tuberosum*	UK, 1957
*Pectobacterium carotovorum* subsp. *carotovorum* NCPPB 1848	*Cattleya* sp.	Brazil, 1966
*Clavibacter michiganensis* subsp. *sepedonicus* NCPPB 4053	*Solanum tuberosum*	Sweden, 1994
*Pseudomonas* sp. 183/03-2	*Pyrus communis*	Slovenia, 2003
*Escherichia coli* GSPB 48	NA	NA
*Brenneria alni* CFBP 3923	*Alnus cordata*	Italy, 1990
*Brenneria quercina* NCPPB 1852	*Quercus* sp.	USA, 1966
*Proteus vulgaris*	environmental bacteria	NA
*Pantoea ananatis* 940/18-11	*Zea mays*	Slovenia, 2011
*Pantoea agglomerans* 363/18-2	*Zea mays*	Slovenia, 2018
*Erwinia amylovora* 106/13-1	*Pyrus* sp.	Slovenia, 41395
*Serratia liquefaciens* 103/14-8	*Lycopersicon esculentum*	Slovenia, 41760
*Enterobacter* sp. NCCPB 4168	NA	NA, 2001
*Burkholderia gladioli* pv. *gladioli* NCPPB 1891	*Gladiolus* sp.	NA, 1966
*Acidovorax avenae* subsp. *cattleyae* NCPPB 4196	*Phalaenopsis* sp.	Brazil, 2000
*Paraburkholderia caryophylli* NCCPB 353	NA	USA, 1954
*Bacillus polymyxa* NCCPB 4162	*Solanum tuberosum*	France, 2001
*Ralstonia solanacearum* 12/19-3	*Solanum tuberosum*	Slovenia, 2019

### 2.3 Samples and sample preparation

#### 2.3.1 Analytical specificity

Suspensions of target and non-target bacteria (Table 2) were prepared from overnight cultures in 10 mM phosphate buffer (PB; 1.07 g Na_2_HPO_4_, 0.4 g NaH_2_PO_4_ × 2H_2_O per liter of water, pH 7.2) to an approximate concentration of 10^6^ cells/mL (Densitometer DEN-1, Biosan). Inclusivity was determined on five *D. fangzhongdai* isolates from three different geographical regions (Asia, America, Europe) and 3 different host niches (herbaceous plants, trees, water). Exclusivity was determined on other *Dickeya* spp. (17 isolates), selected bacteria from *Enterobacteriaceae* family (15 strains), and bacteria colonizing the same host plant niches (5 isolates) as the target bacteria (Table 2).

#### 2.3.2 Analytical sensitivity

A *D. fangzhongdai* B16 and JS5T bacterial suspension with concentration of 10^7^ cells/mL was prepared in a 10 mM PB with 30% (V/V) glycerol. DNA was extracted and standard curves were prepared by 10-fold dilutions in TE buffer (Sigma-Aldrich, Merck, Germany) with the addition of salmon sperm DNA (25 μg/mL).

#### 2.3.3 Diagnostic sensitivity

Plant extracts were prepared from relevant asymptomatic plants, namely orchids (genus *Phalaenopsis*), potato (*Solanum tuberosum*, cultivar Carrera), and apple (*Malus domestica*). For the preparation of *Phalaenopsis* extracts, leaf material was collected in July 2019 and surface sterilized with 70% ethanol. One gram of leaf tissue was macerated in 3.5 mL of sterile 10 mM phosphate buffered saline (10 mM PBS; 1.08 g Na_2_HPO_4_, 0.4 g NaH_2_PO_4_ × 2H_2_O, 8 g NaCl, 1 L distilled water, pH 7.2). The supernatant was separated from the plant tissue by pipetting. A field sample of an asymptomatic potato plant, cultivar Carrera, was collected in July 2016. The surface of the sampled plant stems was cleaned and surface sterilized with 70% ethanol. The asymptomatic potato stem was cut into smaller pieces (approximately 2 cm size) and covered with sterile 10 mM PBS buffer, vortexed, and incubated for several minutes (up to 20 min) at room temperature. The supernatant was separated from the plant tissue by pipetting. An apple extract was prepared from *Malus domestica* asymptomatic plant material collected in July 2018. The sample material consisted of twigs that were surface sterilized with 70% ethanol. Vascular tissue was scraped from sampled twigs and covered with sterile 10 mM PBS containing 0.1% Tween 20, vortexed, and incubated with shaking for 90 min at room temperature. The supernatant was then separated from the plant tissue and centrifuged at 1,500 *g* for 10 min, transferred to a new tube and centrifuged at 7,000 *g* for 20 min. The pellets were suspended in 2 mL of 10 mM PBS.

The health status of plant extracts was confirmed with real-time PCR analysis using generic *Dickeya* spp. assay (Pritchard et al., 2013b).

Surface water was sampled from the Pivka River in a western part of Slovenia in August 2017. Temperature and pH of the water at the time of sampling were 22°C and 7, respectively. One liter of water was aliquoted to 250 mL and centrifuged for 20 min at 10,000 *g* at 4–10°C. Pellets were resuspended in 1 mL of 10 mM PB buffer. The absence of *Dickeya* spp. in the surface water extract was confirmed by real-time PCR analysis using generic *Dickeya* spp. assay (Pritchard et al., 2013b).

Standard curves of *Dickeya fangzhongdai* B16 in plant extracts and surface water extract were prepared by mixing bacterial suspensions with aliquots of extracts to final concentrations ranging from 10^7^ to 10^1^ cells/mL of plant or surface water extract.

#### 2.3.4 Retrospective survey

A retrospective survey was performed on the collection of DNA extracts from sample material selected as described in Section “2.1 Selection of host plants and niches relevant for *D. fangzhongdai* survey.” Potato samples, *Malinae* samples, and surface water samples previously obtained in diagnostic activity in the years 2017–2021 were included in the survey. Altogether, 278 plant samples were analyzed, consisting of 130 potato samples, 148 *Malinae* samples and 53 surface water samples.

##### 2.3.4.1 Samples of potato plants and tubers

Samples of potato plants and tubers with soft rot symptoms were analyzed. The surface of the sampled plants was cleaned, and surface sterilized with 70% ethanol. Symptomatic material was covered with sterile 10 mM PBS buffer, vortexed, and incubated for several minutes (up to 20 min) at room temperature. The supernatant was separated from plant tissue. DNA was extracted as described in Section “2.3 Samples and sample preparation.” Extracted DNA was stored below −15°C until analysis. Potato samples comprised of potato plants with soft rot symptoms (119 samples) and potato tubers with soft rot (11 samples).

##### 2.3.4.2 *Malinae* samples

Tree samples (from the *Malinae* subtribe) were surface sterilized with 70% ethanol. Vascular tissue was scraped from sampled twigs and covered with sterile 10 mM PBS containing 0.1% Tween 20, vortexed, and incubated for 90 min at room temperature. The supernatant was then separated from the plant tissue and centrifuged at 1,500 *g* for 10 min, transferred to a new tube and centrifuged at 7,000 *g* for 20 min. The pellets were suspended in 2 mL of 10 mM PBS. DNA was extracted as described in Section “2.4 DNA extraction and purification.” Extracted DNA was stored below −15°C until analysis. The *Malinae* samples were included apple samples (84 samples), pear samples (8 samples) and Asian pear sample (1 sample) with fire blight symptoms, and mixed *Malinae* samples (55 samples) sampled for latent testing to fire blight.

##### 2.3.4.3 Surface water samples

Fifty diagnostic samples of surface water were collected from different freshwater. All samples were collected in summer, and the temperature and pH of the water at the sampling site were recorded (Supplementary Table 1). Samples were analyzed as follows: one liter of water was aliquoted to 250 mL and centrifuged for 20 min at 10,000 *g* at 4–10°C. Pellets were resuspended in collectively 1 mL of 10 mM PB buffer. DNA was extracted as described in Section “2.3 Samples and sample preparation.” Extracted DNA was stored below −15°C until analysis.

### 2.4 DNA extraction and purification

DNA was extracted from 100 μL aliquots of pure bacterial suspensions, spiked plant extracts, spiked surface water, field plant samples, and surface water samples using magnetic beads-based DNA extraction on QuickPick SML Plant DNA kits (BioNobile, Finland), according to Pirc et al. (2009), with the minor modification of using 440 μL lysate in the downstream purification.

DNA used for analytical specificity was extracted from 500 μL of pure bacterial suspension in PB buffer using heat lysis. Samples were incubated at 95°C for 10 min in a thermoblock, and then immediately put on ice for 3 min. After centrifugation for 1 min at 6,000 rpm supernatant was collected.

### 2.5 Real-time PCR assay design

A *D. fangzhongdai* specific real-time PCR assay was designed according to Alič et al. (2022). Unique diagnostic markers of *D. fangzhongdai* strains were identified by RUCS (Thomsen et al., 2017). A positive dataset comprised of 10 *D. fangzhongdai* genomic sequences was compared to a negative dataset of 39 *Dickeya* spp. genomic sequences, including *D. solani*, *D. dadantii*, *D. dianthicola*, *D. chrysanthemi*, *D. undicola*, *D. aquatica*, *D. zeae*, and *D. paradisiaca* (Supplementary Table 2). The complete genome sequence of *D. fangzhongdai* ND14b was selected as the positive reference genome.

Specificity of the identified unique sequences was confirmed by Blastn (Altschul et al., 1990) analysis against the whole GenBank database. Altogether, nine suitable unique sequences of sufficient length (above 100 bp) were identified. Primers and hydrolysis probes for real-time PCR were designed using Primer Express version 2.0 (Applied Biosystems). The quality of the designed assays was evaluated *in silico* by OligoAnalyzer Tool (IDT) and Blastn (Altschul et al., 1990), and experimentally.

The optimal assay, assay Df_tr (Table 3; Supplementary Table 3), designed against a transcriptional regulator gene (Dickeya_fangzhongdai_ND14b.0976; GenBank locus tag LH89_04605), was selected for validation (Alič et al., 2019). Assays with poor performance or those targeting hypothetical genes or genes of extrachromosomal origin were omitted from further analysis.

**TABLE 3:** Primers and probes used in real-time PCR assays designed and evaluated in this study.

Assay	Name	Sequence (5′-3′)	Amplicon length
Df_tr	Df_tr_F	GGCCGCGTCTAT GTTCTCA	76 bp
	Df_tr_P	FAM-ACTGCATGGCGTCAATAT TTCCCCC-BHQ1	
	Df_tr_R	ACATACATTTGACACCGT CATATTTGT	

### 2.6 Setup of the real-time PCR experiment

Real-time PCR reactions were performed on a QuantStudio 7 (Applied Biosystems, Thermo Fisher) using universal cycling conditions (2 min at 50°C, 10 min at 95°C, followed by 45 cycles of 15 s at 95°C and 1 min at 60°C, with 1.6°C/s ramping speed) according to the PCR Master Mix manufacturer’s recommendations. The reaction volume of 10 μL contained, in final concentrations: 1x TaqMan™ Universal PCR Master Mix (Applied Biosystems, Thermo Fisher), 900 nM primers (Eurofins), 200 nM probe (Eurofins), and 2 μL DNA. The QuantStudio™ real-time PCR Software 1.3 and 1.6 (Applied Biosystems, Thermo Fisher Scientific) were used for fluorescence acquisition and calculation of the threshold cycles (Cq). The baseline was set automatically, and the fluorescence threshold was set manually to intersect with the linear part of the amplification curves of all real-time PCR assays.

Analysis parameters in Df_tr validation procedure included the automatic baseline setting, and the fluorescence threshold set manually to 0.05.

Amplification of the plant endogenous sequence COX was used as an extraction and amplification control [COX; Weller et al. (2000), forward primer and probe and Mumford et al. (2004), reverse primer)]. real-time PCR assays for non-specific detection of *Dickeya* spp., assay ECH (Pritchard et al., 2013b) was used as a control for presence of *Dickeya* spp. The standard curves prepared by mixing target bacteria and plant extracts were used to determine analytical sensitivity of the novel assay and the real-time assay described by Tian et al. (2020). Fluorescence thresholds for those assays were manually set to 0.1, 0.1, and 0.06 for COX, ECH, and Df_tr assay, respectively. A reaction was interpreted as positive if it produced an amplification curve and a fluorescence signal that exceeded the threshold.

Positive amplification controls and negative amplification controls were included in every real-time PCR experiment for each assay.

### 2.7 Validation of *D. fangzhongdai* specific real-time PCR assays

#### 2.7.1 Analytical specificity and selectivity

The analytical specificity of the real-time PCR assay was tested *in silico* by Blastn (Altschul et al., 1990) and experimentally by amplification of five target *D. fangzhongdai* strains and 37 non-target strains, including strains from eight different *Dickeya* genera (Table 2). Selectivity of the assay was tested on relevant plant matrixes, namely plant extracts from orchid plants, potato plants, and apple tree bark scrapings, free of disease symptoms.

#### 2.7.2 Analytical and diagnostic sensitivity

Analytical sensitivity was determined in dilutions of DNA from pure cultures of *D. fangzhongdai* B16 and *D. fangzhongdai* JS5*^T^*, and diagnostic sensitivity was determined on standard curves of *D. fangzhongdai* B16 in plant extracts of *Phalaenopsis*, potato and apple, and surface water. Each standard curve was analyzed in triplicate. The following control systems were used to assure reliability of results and provide further information on method performance: (i) use of undiluted and diluted (1:10 in molecular grade water) DNA extracts from spiked plant extracts and surface water, and (ii) amplification of plant endogenous sequence as an extraction and amplification control (COX) (Weller et al., 2000; Mumford et al., 2004).

The limit of detection LOD_95_ was defined as the target amount giving positive results with 95% confidence and was calculated using drc package in R (Ritz and Strebig, 2016; R Core Team, 2021). The slope (k) of the linear regression line between logarithmic values of cell numbers (independent variable) and Cq values (dependent variable) was used to calculate the amplification efficiency, *E* = (10[-1/k])^–1^, where a value of one corresponds to 100% amplification efficiency (Pfaffl, 2001). The dynamic range, *i.e*., the range of concentrations for which Cq values were in linear relationship with logarithms of concentrations, was determined by visually exploring the slope across sections of the Cq values × log concentration plot.

Performance of the developed real-time PCR Df_tr assay was compared to real-time PCR assay described by Tian et al. (2020).

## 3 Results

### 3.1 Selection of host plants and niches relevant for *D. fangzhongdai* survey

*Dickeya fangzhongdai* isolates collected from reports in publications and the GenBank database are shown in Table 1. The majority of the species (87%) were reported from Asia. The reported isolates were predominantly isolated from soft rot symptoms on orchids (48%) and taro plants (20%). Six isolates (6%) were reported to be isolated from Asian pear trees, causing bleeding canker disease and 5 isolates (5%) were isolated from water sources. Overall, 80% of isolates were isolated from monocot plants, suggesting that *D. fangzhongdai* might have preference based on cotyledon types. Since *D. fangzhongdai* species description is relatively new, it is very likely that isolates found before the species description were assigned only to *Dickeya* spp. level, as was the case for NCPPB 3274. Therefore, the true list of *D. fangzhongdai* isolates is likely to be far more substantial. For example, it was indicated that *Dickeya* spp. isolates from several host plants described by Suharjo et al. (2014) correspond to *D. fangzhongdai* (Alič et al., 2017a,^2018^).

*Dickeya fangzhongdai* isolates with known whole genome sequence (Table 1) share above 96% average nucleotide identity (ANI) and above 86% coverage, regardless of the geographical origin or host.

Based on the literature search and previous experiences, the survey was focused on plants of agricultural importance (e.g., potato and members of the *Malinae* subtribe) and water samples. The latter give broader environment representation compared to individual plant samples.

### 3.2 *Validation of the real-time PCR Df_tr assay*

#### 3.2.1 Analytical specificity and selectivity

The real-time PCR assay Df_tr, targeting a transcriptional regulator containing an amidase domain and an AraC-type DNA-binding HTH domain, was found to be specific for detection of *D. fangzhongdai* species. The assay exhibited 100% inclusivity (5/5 isolates) since all *D. fangzhongdai* isolates were reliably detected regardless of their host or geographical origin. Moreover, no cross reactivity with any of the tested non-target bacteria (37 isolates) was observed, meaning that the test is 100% exclusive to *D. fangzhongdai*.

Lastly, no non-specific amplification was obtained from healthy plant matrices, therefore selectivity of the assay was determined to be 100% on tested matrices, namely potato, orchid, and apple tree.

#### 3.2.2 Analytical sensitivity

Analytical sensitivity was determined on DNA standard curves of two different *D. fangzhongdai* isolates, B16 and JS5*^T^*, from different environmental niches. The newly developed assay showed high analytical sensitivity (Table 4) with LOD_95_ below 10^4^ cells/mL of plant extracts in all three matrices. The performance characteristics of the real-time PCR Df_tr assay were very similar to the real-time PCR described by Tian et al. (2020) (Supplementary Table 4). Both assays gave almost identical results tested on DNA standard curves.

**TABLE 4:** Performance characteristics of real-time PCR Df_tr assays evaluated on bacterial suspension, spiked plant matrices and spiked surface water.

	Dynamic range (cells/mL)^1^		Linear regression^2^			LOD_95_^3^		
	**From**	**To**	**Slope (k)**	**R^2^**	**E**	**Log. conc [log(cells/ml)]**	**Cells/** **mL**	**Residual error**
**DNA standard curve**								
*D. fangzhongdai* B16	10^4^	10^7^	−3.6	1.00	0.91	3.7	5164	6.22 × 10^–02^
*D. fangzhongdai* JS5^T^	10^3^	10^7^	−3.4	1.00	0.98	2.5	311	7.21 × 10^–10^
**Spiked plant matrix**								
Potato plant	10^4^	10^6^	−3.3	0.99	1.01	2.5	311	7.21 × 10^–10^
Orchids	10^3^	10^7^	−3.5	1.00	0.93	2.5	325	3.03 × 10^–02^
Apple tree	10^4^	10^7^	−3.6	1.00	0.91	3.4	2275	3.03 × 10^–02^
**Spiked water**								
Surface water	10^4^	10^7^	−3.0	0.99	1.14	3.6	3776	3.03 × 10^–02^

^1^The range of concentrations for which Cq values were in linear relationship with logarithms of concentrations.

^2^Linear regression of all positive samples of Cq values against logarithmic number of *D. fangzhongdai* cells; k: slope of the determined linear regression line; R2: average square regression coefficient; E: efficiency of amplification calculated from k.

^3^LOD_95_: limit of detection was defined as the target amount giving positive results with 95% confidence.

#### 3.2.3 Diagnostic sensitivity

**FIGURE 1:**
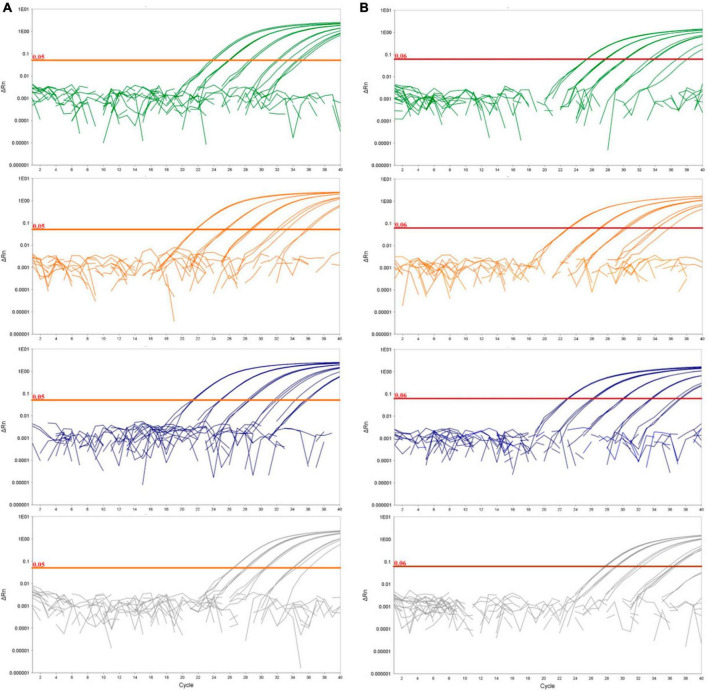
Logarithmic amplification curves of *D. fangzhongdai* DNA extracted from spiked plant matrices and spiked surface water for the novel real-time PCR assay Df_tr **(A)** and assay described by Tian et al. (2020) **(B)**. The curves show bacterial standard curves prepared in potato matrix (green curves), in apple matrix (orange curves), in orchid matrix (blue curves) and in surface water (gray curves). The threshold line for the real-time PCR assay Df_tr is shown in orange and for Tian et al. (2020) in red.

Diagnostic sensitivity was determined on spiked plant matrices and surface water. No inhibition of amplification could be observed in any of the matrices tested; however, the sensitivity of the assay varied from matrix to matrix, suggesting a matrix effect on DNA extraction procedure (Figure 1). Of all the plant matrices tested, sensitivity was most affected by the apple tree matrix, with an LOD_95_ of 2,275 cells/mL (Supplementary Figure 1) compared to potato and orchid matrices with LOD_95_ in the range of 311 – 325 cell/mL of sample. The diagnostic sensitivity of the assay was slightly lower in surface water than in plant matrices. The LOD_95_ was of 3,776 cells/mL of sample (Supplementary Figure 1). However, the performance characteristics of the matrices tested were not significantly different (Table 4). The Cq values were consistently below 37 at the detection limit where all parallel reactions were positive for all samples tested. Inter-run repeatability was high for all the samples and matrices tested, with coefficients of variation of Cq values below 7% within the dynamic range. The performance characteristics of the real-time PCR Df_tr assay were better compared to performance characteristics of the real-time PCR described by Tian et al. (2020) in all spiked matrices (Supplementary Table 4).

**FIGURE 2:**
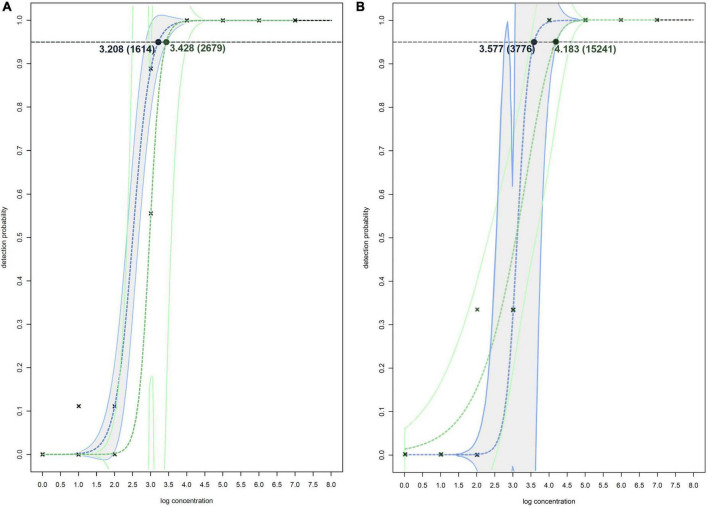
Non-linear modeling of probability of detection on spiked plant matrixes **(A)** and spiked surface water **(B)** for Df_tr real-time PCR assay (shown in blue and gray) and real-time PCR assay described by Tian et al. (2020) (shown in green). The concentrations shown are expressed as log(cells/mL of plant extract), and in the brackets as cells/mL of plant extracts. The model used for both assays on spiked plant matrixes **(A)** is two-parameter log-logistic function (LL.2), and models used on spiked surface water **(B)** is log-logistic function (LL.2) for Df_tr real-time PCR assay and two-parameter Weibull function (W2.2) for real-time PCR assay described by Tian et al. (2020). The dotted line denotes 95% probability of detection.

The greatest difference in sensitivity between assays was observed in the plant matrices. The LOD_95_ of the Df_tr assay was 311 and 325 cells/mL, compared to the real-time PCR described by Tian et al. (2020), which had LOD_95_ of 2,275 and 2,438 cells/mL, for potato and orchid matrices, respectively. Nonetheless, the difference in sensitivity was less pronounced for the apple tree matrix (LOD_95_ of 2,275 for the Df_tr assay and 3,776 for the real-time PCR described by Tian et al. (2020). A similar difference in sensitivity was observed in surface water. The Df_tr assay showed higher sensitivity, with LOD_95_ of 3,776 cells/mL than the real-time PCR described by Tian et al. (2020) with LOD_95_ of 15,241 cells/mL (Figure 2). Overall, the sensitivity of Df_tr assay was better than the real-time PCR described by Tian et al. (2020) in plant matrices and surface water (Figure 2). In addition, fluorescence (ΔRn; Figure 1) was consistently higher in the Df_tr assay compared to real-time PCR described by Tian et al. (2020).

No false positives were observed for Df_tr. As expected, only samples with a bacterial concentration below LOD_95_ gave false negative results when compared with their known health status (Table 5). Accuracy of the test based on spiked samples was 79% and diagnostic sensitivity 76%.

**TABLE 5:** Contingency table for real-time PCR Df_tr calculated on spiked plant samples.

		Known status		
		**Infected**	**Non-infected**	**Total**
Result of test	pos	TP^a^	FP^b^	TP + FP
		16.0	0.0	16.0
	neg	FN^c^	TN^d^	FN + TN
		5.0	3.0	8.0
	total	TP + FN	FP + TN	N^e^
		21.0	3.0	24.0

If a sample was spiked with *D. fangzhongdai* B16 suspension, its health status was considered “infected” even if the concentration was below the expected LOD. The table combines the results for all 3 tested plant matrices.

^a^True positive;

^b^False positive;

^c^False negative;

^d^True negative;

^e^Total sample count.

**FIGURE 3:**
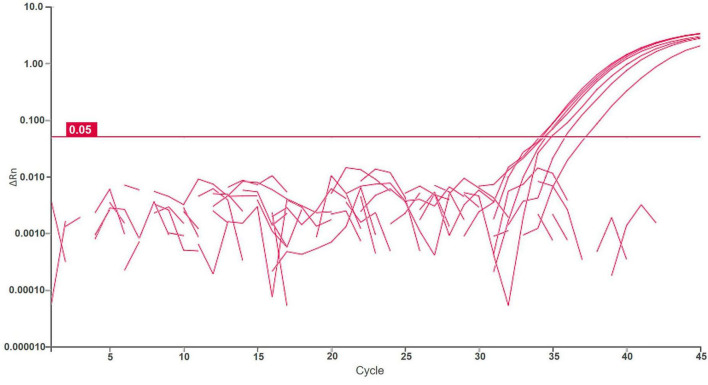
Logarithmic amplification curves of positive surface water samples of real-time PCR Df_tr. Threshold line is shown in magenta. Each sample was analyzed in duplicates, and it was considered positive if at least one reaction was positive.

Samples of potato plants and tubers, and samples of *Malinae* trees were tested for a general presence of *Dickeya* spp. and *D. fangzhongdai* strains. The retrospective assay did not confirm the presence of *D. fangzhongdai* in any of the tested plant samples. However, 12% (16/130 samples) of tested samples with soft rot symptoms were positive using a non-specific *Dickeya* spp. real-time assay (Pritchard et al., 2013b), indicating the presence of other *Dickeya* species in potato. Similarly, no *D. fangzhongdai* nor other *Dickeya* spp. were detected in any of the samples of *Malinae* members. The *Malinae* samples were collected from trees that are of economic importance in the Slovenian environment, therefore the majority of the samples represent the genera *Pyrus* and *Malus*. The general presence of *Dickeya* spp. was confirmed in samples of surface water using real-time PCR. *Dickeya* spp. were detected in 70% (35 out of 50) surface water samples in low concentrations (32 ≤ Cq ≤ 39). In 6 of these samples, we also detected *D. fangzhongdai*, in 4 samples from August 2018 and 2 samples from August 2021. In all samples, concentrations were relatively low (34 ≤ Cq ≤ 38; Figure 3), close to the limit of detection of the assay (LOD_95_ of 3,776 cells/mL of sample water extract; Figure 2). The sample was considered positive if at least one reaction produced a signal above threshold and a characteristic amplification curve was present.

Positive water samples were from different freshwater sources and of different types (Table 6). The samples were collected from different parts of Slovenia, but most samples had slightly acidic pH of 6 (4 of 6 samples). The temperature of the water ranged from 17°C to 28.5°C, measured at the sampling site.

**TABLE 6:** Metadata of the water samples positive presence of *Dickeya* spp. (real-time PCR ECH) and *D. fangzhongdai* (real-time PCR Df_tr).

Sampling location, Year	Type of surface water	pH	Water temperature [°C]
Manče, 2021	Spring	7.0	19.0
Dobruška vas, 2021	River	6.0	18.0
Gradišče, 2018	Lake	6.0	27.0
Radehova, 2018	Lake	6.5	28.5
Vanganel, 2018	Stream	6.0	17.0
Ajdovščina, 2018	Stream	6.0	24.0

## 4 Discussion

In this study, a real-time PCR assay was developed for specific detection of *D. fangzhongdai*, along with its validation in matrices of orchids, potatoes, and *Malinae* members. The assay was used in a retrospective survey of relevant ecological niches in Slovene environments.

Designed assay exhibited very good performance characteristics in the validation, which proves its suitability for the detection of *D. fangzhongdai* with 100% inclusivity and exclusivity and good analytical and diagnostic sensitivity. Diagnostic sensitivity of the test ranged from 1 to 10 cells per reaction (LOD_95_), showing that the sensitivity of the assays is close or equal to the theoretical sensitivity of the method (Kralik and Ricchi, 2017). No inhibition of the real-time PCR reaction could be observed in any of the tested plant matrices and the reaction efficiency was close to optimal. Compared to the real-time assay described by Tian et al. (2020), the new test exhibited better diagnostic sensitivity in samples that contained plant matrices, showing better suitability for diagnostic purposes.

*Dickeya fangzhongdai* is the first known member of *Dickeya* spp. that causes disease not only on herbaceous plants, but also on trees. The majority of *Dickeya fangzhongdai* isolates originate from Asia, and few occurrences of *D. fangzhongdai* have been reported in Europe or America. This species has not yet been found to be associated with any significant plant disease in Europe’s open environment. It has been isolated from asymptomatic potato tubers in the Netherlands, but never from symptomatic plants in farmers’ fields in Europe (van der Wolf et al., 2022). Water sources seem to be an alternative habitat for *Dickeya* spp., as three species, namely *D. aquatica*, *D. undicola* and *D. lacustris*, are limited to water habitats and many others, including *D. fangzhongdai*, were also isolated from various water sources (Pritchard et al., 2013a; Parkinson et al., 2014; Alič et al., 2018; Hugouvieux-Cotte-Pattat et al., 2019; Oulghazi et al., 2019). Furthermore, presence of *D. fangzhongdai* was indirectly confirmed in wastewaters in Slovenia by isolation of *D. fangzhongdai* specific bacteriophages (Alič et al., 2017b). The results of the screening test performed in this study correspond with the described findings. However, the importance of water as an ecological niche is not yet understood. It is not known whether water presents a transmission source or only transient ecological niche that the bacteria is able to persist in. Nevertheless, virulence genes and genes involved in virulence regulation are also conserved in isolates from water (Alič et al., 2019).

In the screening test of potato plants and tubers with and without soft rot symptoms, and trees from the *Malinae* subtribe, *D. fangzhongdai* could not be detected in the tested samples. In Asian pear trees, bark tissue has been shown to be affected by *D. fangzhongdai*, therefore vascular tissue from twigs was selected as sample material for *Malinae* samples (Tian et al., 2016; Chen et al., 2020). However, it is not known which tissue would be most suitable for testing asymptomatic trees for the presence of *D. fangzhongdai*. Sixty-five of the surface water samples tested in this study were positive for presence of *Dickeya* spp., and 11% of those samples also contained *D. fangzhongdai*. *D. fangzhongdai* was present at low concentration, and the limit of detection (LOD_95_) of the assay in surface water is 3,776 cells/mL sample water extract. Other *Dickeya* spp. was detected in some potato samples with soft rot symptoms, however its prevalence is approximately 5 times lower compared to water samples. Based on the results, *Dickeya* spp. including *D. fangzhongdai* have not yet entered the agricultural environment but is present at low concentrations in some water sources in Slovenia.

Repeated reports of *D. fangzhongdai* outbreaks in Asia indicate that the pathogen is posing a threat to cultivation of various crops, ornamental plants and trees (Tian et al., 2016; Zhang et al., 2018; Jaffar et al., 2019; Shen et al., 2019; Tsai et al., 2019; Balamurugan et al., 2020; Choi et al., 2021; Huang et al., 2021; Wang et al., 2021). There have been no reports of *D. fangzhongdai* outbreaks or infections of plants in the open environment in Europe, however due to lack of specific testing for *D. fangzhongdai* species, isolates can be overlooked or assigned to *Dickeya* spp. The newly developed real-time PCR is reliable, sensitive and adequately validated, and therefore a suitable detection test for *D. fangzhongdai* detection, identification, and monitoring. Based on the results of the retrospective survey, *D. fangzhongdai* seems to be present in some water sources in Slovene environment. Presence of *D. fangzhongdai* was not confirmed in any tested plant species, however, its persistence cannot be excluded from hosts that were not included in this study. Specific testing for *D. fangzhongdai* presence and accordingly implementing preventive measures, is currently the only mechanism to prevent establishment of the species in new environments and environments in which the species had been sporadically detected.

Since the beginning of the 21st century the most detrimental *Dickeya* spp. for in European agriculture was *D. solani*. In 2012 it was listed among the 10 most important bacterial pathogens because of its sudden clonal spread and impact on the potato industry under higher temperatures (Mansfield et al., 2012). The pathogen was first isolated in 2005 and then in 2009 (Sławiak et al., 2009) but was recognized as a species only in 2014 (van der Wolf et al., 2014). However, in more recent studies it was shown that *D. solani* was present in potato more than a decade before the first reported outbreak. The early strains are genetically very close to the epidemic clones isolated during the 2000s outbreaks. Potentially aggressive *D. solani* strains in potato seeds were already present in the last century (Pédron et al., 2021), therefore it does not seem that genetics played an exclusive role in promoting pathogenicity, but rather an additional factor to the environmental conditions. Based on the reports, *D. fangzhongdai* seems to be as or even more aggressive than *D. solani* (Alič et al., 2017a; van der Wolf et al., 2022). Even if there is currently no association of *D. fangzhongdai* with any host in the open European environment, it is highly likely that the pathogen will not have a problem finding a host in favorable environmental conditions. Previous experience with *D. solani* showed that sporadic detection of such an aggressive pathogen in the environment might be a warning sign supporting the need for specific surveying of *D. fangzhongdai*.

## Data availability statement

The datasets presented in this study can be found in online repositories. The names of the repository/repositories and accession number(s) can be found in the article/Supplementary material.

## Author contributions

TD and ŠA: conceptualization, methodology, validation, and funding acquisition. ŠA and KB: formal analysis, investigation, and data curation. ŠA, KB, and TD: resources. ŠA: writing—original draft preparation and visualization. TD: writing—review and editing, supervision, and project administration. All authors have read and agreed to the published version of the manuscript.
