# Supplementary material for: Retrospective survey of Dickeya fangzhongdai using a novel validated real-time PCR assay
Source: Front Microbiol. 2024 Feb 13;14:1249955. doi: 10.3389/fmicb.2023.1249955 (PMC10896844; doi:10.3389/fmicb.2023.1249955)
Supplement: Supplementary file 1 [file Data_Sheet_1.docx]

Supplementary Material

Retrospective survey of *Dickeya fangzhongdai* using a novel validated real-time PCR assay

Špela Alič^1^, Katarina Bačnik^1^ and Tanja Dreo^1,*^

^1^National Institute of Biology, Vecna pot 111, 1000 Ljubljana, Slovenia

*** Correspondence:**Tanja Dreo
tanja.dreo@nib.si

# Supplementary Tables

**Supplementary Table 1.** Metadata of the water samples

| **Sampling location** | **Sampling time** | **Type of surface water** | **pH** | **Water**  **temperature [°C]** |
| --- | --- | --- | --- | --- |
| Lahovče | August 2017 | stream | 7.0 | 22.0 |
| Stična | August 2017 | stream | 7.0 | 20.0 |
| Strahinj | August 2017 | stream | 7.0 | 21.0 |
| Turnišče | August 2017 | NA | 6.0 | 25.0 |
| Mihovci | August 2017 | stream | 6.0 | 16.0 |
| Podgorje pri SG | August 2017 | stream | 7.0 | 14.0 |
| Studence | August 2017 | stream | 7.0 | 19.5 |
| Smrje | August 2017 | river | 6.0 | 21.0 |
| Pivka | August 2017 | river | 7.0 | 22.0 |
| Šutna | August 2017 | stream | 7.0 | 18.0 |
| Vangel | August 2018 | stream | 6.0 | 24.0 |
| Zgornji Brnik | August 2018 | stream | 7.0 | 15.0 |
| Ajdovščina | August 2018 | stream | 6.0 | 17.0 |
| Križevci | August 2018 | artificial lake | 6.0 | 29.5 |
| Juršinci | August 2018 | stream | 7.0 | 24.0 |
| Kraščci | August 2018 | lake | 6.0 | 27.0 |
| Radehova | August 2018 | lake | 6.5 | 28.5 |
| Hrvaški brod | August 2018 | stream | 7.0 | 21.0 |
| Kanižarica | August 2018 | river | 7.0 | 20.0 |
| Gradišče | August 2018 | lake | 6.0 | 27.0 |
| Trpačne | July 2019 | river | 6.0 | 20.0 |
| Pivka | July 2019 | stream | 7.0 | 23.0 |
| Ljubljana | July 2019 | stream | 7.0 | 22.0 |
| Gorenja vas | July 2019 | NA | 7.0 | 19.0 |
| Moste pri Komendi | July 2019 | stream | 7.0 | 21.0 |
| Tešanovci | August 2019 | stream | 8.5 | 21.0 |
| Cerkvenjak | August 2019 | lake | 7.0 | 26.0 |
| Slovenska Bistrica | August 2019 | stream | 6.0 | 19.0 |
| Dobrovnik | August 2019 | lake | 5.5 | 24.0 |
| Znojle pri Krki | August 2019 | stream | 8.0 | 19.0 |
| Črnolica | August 2020 | river | 5.5 | 19.0 |
| Strahinj | August 2020 | stream | 7.0 | 21.0 |
| Zgornje Roje | August 2020 | stream | 6.0 | 17.0 |
| Vosek | August 2020 | lake | 7.0 | 18.0 |
| Metava | August 2020 | stream | 7.0 | 16.0 |
| Veliko Mlačevo | August 2020 | stream | 6.0 | 14.0 |
| Mala Kostrevnica | August 2020 | stream | 6.0 | 16.0 |
| Malo Ubeljsko | August 2020 | stream | 7.0 | 21.0 |
| Podbreže | August 2020 | stream | 7.0 | 19.0 |
| Okroglo | August 2020 | river | 7.0 | 15.0 |
| Jablje | August 2021 | stream | 6.0 | 15.5 |
| Vogrsko | August 2021 | stream | 6.0 | 18.0 |
| Komenda | August 2021 | stream | 6.0 | 18.5 |
| Sevnica | August 2021 | river | 6.0 | 17.5 |
| Dobruška vas | August 2021 | river | 6.0 | 18.0 |
| Dolenja vas | August 2021 | stream | 6.0 | 15.0 |
| Legen | August 2021 | stream | 6.0 | 14.0 |
| Gortina | August 2021 | river | 6.0 | 13.0 |
| Fram | August 2021 | stream | 6.0 | 13.5 |
| Manče | August 2021 | spring | 7.0 | 19.0 |

**Supplementary Table 2**. *Dickeya* spp. strains and their corresponding GenBank accession numbers of the genomes included in the *Dickeya fangzhongdai* specific real-time PCR design.

| **Dickeya strain** | | **GenBank/NCBI accession number** |
| --- | --- | --- |
| *Dickeya fangzhongdai* | |  |
|  | NCPPB 3274 | AOOH01000000 |
|  | MK7 | AOOO01000000 |
|  | M074 | JRWY01000000 |
|  | M005 | JSXD01000000 |
|  | Secpp 1600 | CP023484.1 |
|  | JS5 | CP025003.1 |
|  | B16 | JXBN02000000 |
|  | S1 | JXBO02000000 |
|  | PA1 | CP020872.1 |
|  | ND14b | CP009460.1 |
| *Dickeya zeae* | |  |
|  | EC1 | CP006929.1 |
|  | ZJU1202 | AJVN01000000 |
|  | NCPPB 3531 | CM001980.1 |
|  | NCPPB 2538 | CM001977.1 |
|  | MS2 | CP025799.1 |
|  | Ech586 | CP001836.1 |
| *Dickeya* sp. | |  |
|  | NCPPB 569 | CM001975.1 |
| *Dickeya aquatica* | |  |
|  | DW 0440 | CM001983.1 |
|  | CSL RW240 | CM001973.2 |
|  | 174/2 | LT615367.1 |
| *Dickeya undicola* | |  |
|  | 2B12 | JSYG01000000 |
| *Dickeya solani* | |  |
|  | RNS 08.23.3.1.A | CP016928.1 |
|  | RNS 07.7.3B | JWLR01000000 |
|  | RNS 05.1.2A | JWMJ01000000 |
|  | PPO 9134 | JWLT01000000 |
|  | PPO 9019 | CP017454.1 |
|  | IPO 2222 | CP015137.1 |
|  | IFB 0099 | CP024711.1 |
|  | IFB_0221 | PEMZ01000000 |
|  | GBBC 2040 | CM001860.1 |
|  | F012 | PDVN01000000 |
| *Dickeya paradisiaca* | |  |
|  | NCPPB 2511 | CM001857.1 |
| *Dickeya dianthicola* | |  |
|  | S4.16.03.LID | QZDO01000000 |
|  | RNS04.9 | CP017638.1 |
|  | NCPPB 3534 | CM001840.1 |
|  | NCPPB 453 | CM001841.1 |
|  | IPO 980 | CM002023.1 |
|  | GBBC 2039 | CM001838.1 |
|  | DE440 | PJJB01000000 |
| *Dickeya dadantii* | |  |
|  | NCPPB 3537 | CM001982.1 |
|  | NCPPB 2976 | CM001978.1 |
|  | NCPPB 898 | CM001976.1 |
|  | DSM 18020 | CP023467.1 |
|  | 3937 | CP002038.1 |
| *Dickeya chrysantemi* | |  |
|  | NCPPB 3533 | CM001981.1 |
|  | NCPPB 516 | CM001904.1 |
|  | NCPPB 402 | CM001974.1 |
|  | L11 | JSYH01000000 |

**Supplementary Table 3.** Properties of the primers and probe for real-time PCR Df_tr. Both primers and the probe were also analyzed by BLASTn. The best hits for all were the complete genomes of *D. fangzhongdai* with the following alignment characteristics: identity 100%, query coverage 100 %, E-value 9.3 for Df_tr_F; identity 100%, query coverage 100 %, E-value 0.005 for Df_tr_P and 100%, query coverage 100 %, E-value 0.0004.

| Name | Sequence (5'-3') | Length (bp) | GC (%) | Tm (°C) |
| --- | --- | --- | --- | --- |
| Df_tr_F | GGCCGCGTCTATGTTCTCA | 19 | 57.9 | 66.3 |
| Df_tr_P | FAM-ACTGCATGGCGTCAATATTTCCCCC-BHQ1 | 25 | 52.0 | 74.3 |
| Df_tr_R | ACATACATTTGACACCGTCATATTTGT | 27 | 33.3 | 64.9 |

**Supplementary Table 4**. Performance characteristics of the real-time PCR assays for *Dickeya fangzhongdai* described by Tian *et al.* (2020) evaluated on the validation sample set prepared in this study.

|  | **Dynamic range (cells/mL)^1^** | | **Linear regression^2^** | | | **LOD_95_^3^** | | |
| --- | --- | --- | --- | --- | --- | --- | --- | --- |
|  | **from** | **to** | **Slope**  **(k)** | **R^2^** | **E** | **Log conc.**  **(log(cells/mL)** | **cells/**  **mL** | **Residual error** |
| DNA standard curve |  |  |  |  |  |  |  |  |
| *D. fangzhongdai* B16 | 10^4^ | 10^6^ | -3.5 | 1.00 | 0.91 | 3.7 | 5164 | 6.22×10^-02^ |
| *D. fangzhongdai* JS5 | 10^3^ | 10^6^ | -3.6 | 1.00 | 0.90 | 2.5 | 311 | 7.21×10^-10^ |
| Spiked plant matrix |  |  |  |  |  |  |  |  |
| Potato plant | 10^4^ | 10^7^ | -2.7 | 0.99 | 1.31 | 3.4 | 2275 | 3.03×10^-02^ |
| Orchids | 10^3^ | 10^7^ | -3.6 | 1.00 | 0.90 | 3.4 | 2438 | 7.58×10^-02^ |
| Apple tree | 10^4^ | 10^7^ | -3.0 | 0.98 | 1.15 | 3.6 | 3776 | 3.03×10^-02^ |
| Spiked water |  |  |  |  |  |  |  |  |
| Surface water | 10^4^ | 10^7^ | -3.6 | 0.99 | 0.91 | 4.2 | 15241 | 6.89×10^-02^ |

^1^The range of concentrations for which Cq values were in linear relationship with logarithms of concentrations.

^2^Linear regression of all positive samples in a plot of Cq values against logarithmic number of *D. fangzhongdai* cells; k: slope of the determined linear regression line; R2: average square regression coefficient; E: efficiency of amplification calculated from k.

^3^LOD_95_: limit of detection was defined as the target amount giving positive results with 95 % confidence.

# Supplementary Figure

#


**Supplementary Figure 1.** Non-linear modeling of probability of detection for Df_tr real-time PCR assay on spiked plant matrixes, on spiked surface water, and *D. fangzhongdai* DNA standard curves. The concentrations shown are expressed as log(cells/mL). The model used is shown in brackets for each test. Legend: LL2.2 = two-parameter log-logistic function, W1.2 = two parameter Weibull function (type 1), W2.2 = two parameter Weibull function (type 2). The dotted line denotes 95% probability of detection.
